# Comparative evaluation of multiparametric lumbar MRI radiomic models for detecting osteoporosis

**DOI:** 10.1186/s12891-024-07309-0

**Published:** 2024-02-29

**Authors:** Tao Zhen, Jing Fang, Dacheng Hu, Qijun Shen, Mei Ruan

**Affiliations:** 1 Department of Radiology, Hangzhou First People’s Hospital, No.261, Huansha Road, Hangzhou, Zhejiang, 310006 China; 2 Zhejiang Provincial Hospital of Traditional Chinese medicine, Hangzhou, 310006 China

**Keywords:** Osteoporosis, Radiomics, Magnetic resonance imaging, Lumbar spine, Bone mineral density

## Abstract

**Background:**

Osteoporosis is a serious global public health issue. Currently, there are few studies that explore the use of multiparametric MRI radiomics for osteoporosis detection. The purpose of this study was to compare the performance of radiomics features from multiple MRI sequences (T1WI, T2WI and T1WI combined with T2WI) for detecting osteoporosis in patients.

**Methods:**

A retrospective analysis was performed on 160 patients who had undergone dual-energy X-ray absorptiometry(DXA) and lumbar magnetic resonance imaging (MRI) at our hospital. Among them, 86 patients were diagnosed with abnormal bone mass (osteoporosis or low bone mass), and 74 patients were diagnosed with normal bone mass based on the DXA results. Sagittal T1-and T2-weighted images of all patients were imported into the uAI Research Portal (United Imaging Intelligence) for image delineation and radiomics analysis, where a series of radiomic features were obtained. A radiomic model that included T1WI, T2WI, and T1WI+T2WI was established using features selected by LASSO regression. We used ROC curve analysis to evaluate the predictive efficacy of each model for identifying bone abnormalities and conducted decision curve analysis (DCA) to evaluate the net benefit of each model. Finally, we validated the model in a sample of 35 patients from different health care institution.

**Results:**

The T1WI + T2WI radiomics model showed better screening performance for patients with abnormal bone mass. In the training group, the sensitivity was 0.758, the specificity was 0.78, and the accuracy was 0.768 (AUC =0.839, 95% CI=0.757-0.901). In the validation group, the sensitivity was 0.792, the specificity was 0.875, and the accuracy was 0.833 (AUC =0.86, 95% CI=0.73-0.943).The DCA also showed that the combined model had better net benefits. In the external validation group, the sensitivity was 0.764, the specificity was 0.833, and the accuracy was 0.8 (AUC =0.824, 95% CI 0.678-0.969).

**Conclusions:**

Radiomics-based multiparametric MRI can be used for the quantitative analysis of lumbar MRI and for accurately screening patients with abnormal bone mass.

## Background

Osteoporosis is a major global public health issue, but it is frequently underdiagnosed due to low screening rates, unless a significant fragility fracture occurs [1]. Dual-energy X-ray absorptiometry (DXA) is the gold standard for diagnosing osteoporosis, but many patients do not undergo DXA even if they present symptoms [2]. However, lumbar magnetic resonance imageing (MRI) is more commonly used when patients experience low back pain. Previous studies have shown that the lumbar MRI signal is related to bone mineral density, suggesting that this method is useful for detecting osteoporosis [3, 4]. The MRI signal of the bone marrow is determined by its relative amount of bone cells, protein, water and fat. Additional MRI sequences have been developed to provide more information on the bone marrow composition [5–7]. However, traditional imaging methods provide qualitative or semiquantitative results only, while radiomics can extract quantitative features from images to identify additional information and reflect the inherent heterogeneity of lesions. Radiomics has been widely used in disease identification, prognosis evaluation, efficacy evaluation and other aspects [8]. While its use in tumors is prevalent, its application in nonneoplastic diseases has been relatively rare until recently, when there have been increasing studies on musculoskeletal diseases [9–14].

The objective of this study was to explore the possibility of using radiomic features from multiparametric lumbar MRIs to screen patients with osteoporosis.

## Methods

**Fig. 1:**
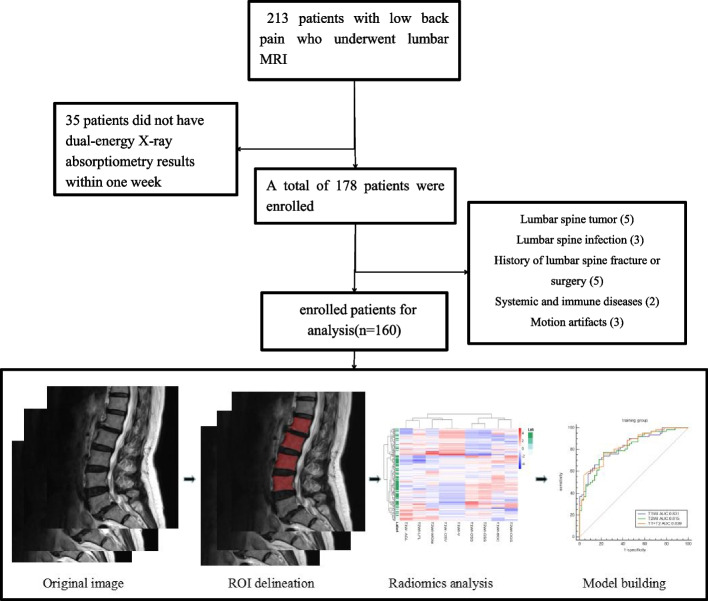
The workflow of the study

Our institutional review board approved this retrospective study, and the requirement for informed consent was waived. The workflow of this study is summarized in Fig .1.

### Patients

The clinical data, including patients’ gender, age, height, weight, and body mass index(BMI), were collected by an orthopedic surgeon. We applied the following inclusion criteria to determine eligibility:

(1) Patients with low back pain lasting more than 3 months and clinical suspicion of osteoporosis. (2) DXA was performed in our hospital, and magnetic resonance imaging was performed within one week. (3) Patients who could cooperate with the MRI examination.

The exclusion criteria were as follows: (1) Patients with a history of lumbar spine fracture. (2) Patients with systemic or immune diseases affecting calcium absorption in bone. (3) Patients with lumbar infection or a lumbar tumor. (4) The MRI images were blurred due to motion artifacts or other reasons. (5) Patients who underwent lumbar spine surgery and had metal implants.

### Imaging examination

The BMD was analyzed through the density of the vertebral body of L1-4 using the American GE company's lunar prdigy DXA, with a tube voltage of 140/100 kV, and a tube current of 2.5 mA. MRI was performed with a 1.5T scanner (Magneton avanto, Siemens AG, Germany), fitted with 8 channel spinal phase control surface coils. The protocol included sagittal T1-weighted images (T1WI) and T2-weighted images (T2WI). The scanning parameters were as follows: T1WI (TR 450 ms TE 9.6 ms), T2WI (TR 2250 ms TE 98 ms), layer thickness (5 mm), layer spacing (0.5 mm), FOV (330 mm×30 mm), and matrix128×128. A total of 10 layers were scanned. The images were exported in Digital Imaging and Communications in Medicine (DICOM) format.

### Evaluation of osteoporosis

The patients were grouped according to the T or Z score obtained by BMD analysis. The women and patients aged ≥50 years were divided into three groups according to their T score: the osteoporosis group (T score ≤-2.5), the low bone mass group (-2.5<T score<-1) and the normal group (T score≥-1). The osteoporosis group and low bone mass group were combined into the abnormal group. Women and patients younger than 50 years were divided into two groups according to the Z score: the normal group (Z score>-2) and abnormal group (Z score≤-2).

### Image processing, feature extraction and screening

Two radiologists with 5 years of experience in musculoskeletal (MSK) imaging manually segmented the L1 to L4 vertebral bodies on sagittal T1WI and T2WI images of lumbar MRI using the uAI Research Portal (United Imaging Intelligence). The region of interest (ROI) was delineated along the edge of the vertebral body, avoiding delineation of the cortical bone. A total of three layers were delineated within the median sagittal plane of each vertebral body and its bilateral sagittal plane.The radiomics module of the Research Portal was used for feature extraction. Features with intraclass correlation coefficients (ICCs) ≥0.75 were retained. Then, the features retained by one of the doctors were standardized by the Z-score normalization algorithm, and the dimension of each feature was reduced by least absolute shrinkage and selection operator (LASSO) regression. The Radscore, defined by the corresponding nonzero coefficients of selected features, was created by a linear combination of weighted features.

### Model establishment and validation

The radiomic model was established based on the radscores, and a receiver operating characteristic (ROC) curve was drawn. The area under curve (AUC) was used to evaluate the predictive efficacy of the model for osteoporosis. Decision curve analysis (DCA) was carried out to evaluate the clinical value of each model on the basis of calculating the net benefit for patients at each threshold probability.

### Statistical analysis

Statistical analysis was conducted with MedCalc (version 19.1) and R software (version 4.1.2). Variables with a normal distribution are shown as the mean ± SD. Variables with a nonnormal distribution are shown as median [iqr]. For continuous clinical variables, Student’s t tests or Mann-Whitney U tests were conducted. For categorical clinical factors, Pearson’s chi-square test or Fisher’s exact test was used. *P* < 0.05 was considered to indicate statistical significance. The Wilcoxon test was used to compare the evaluation efficacy of the rad-scores in the training and validation groups regarding the severity of osteoporosis. The Hosmer–Lemeshow test was used to analyze the fit of the model, and *P* > 0.05 indicated that the model fit was good. The sensitivity, specificity and AUC of the ROC curve were used to evaluate the efficacy of the classification system by Delong’s test.

## Results

### Patients characteristics

A total of 160 patients (24 males and 136 females) aged 33-91 (64.5±11.3) years who underwent DXA and lumbar MR examination at our hospital from January 2017 to August 2021 were retrospectively analyzed. All patients were divided into an abnormal group (*n*=86) and a normal group (*n*=74) according to their BMD determined via DXA. The patients were then randomly stratified at a ratio of 7:3, with the majority used for training (*n*=112 ) and the rest for validation (*n*= 48). Table 1 shows the clinical characteristics of the two groups.

**Table 1:** Clinical characteristics of the training group and the validation group

Variable	Training group(*n*=112)		*P* value	Validation group(*n*=48)		*P* value
	Normal(*n*=50)	Abnormal(*n*=62)		Normal(*n*=24)	Abnormal(*n*=24)	
Age	60.2±12	67.1±9.3	0.000*	61.2±10.2	70.1±9.9	0.011*
Gender (Male/Female)	13/37	3/59	0.001*	6/18	2/22	0.245
Height (cm)	161.7±6.3	157.2±4.9	0.000*	162.6±7.8	157.0±6.7	0.001*
Weight (kg)	63.5±11.4	56.0±7.0	0.002*	62.5±9.2	54.2±7.2	0.004*
BMI	22.7±3.4	23.5±3.2	0.18	23.6±3.3	23.0±3.4	0.511

*Indicates that the difference was statistically significant

### Radiomic analysis

**Fig. 2:**
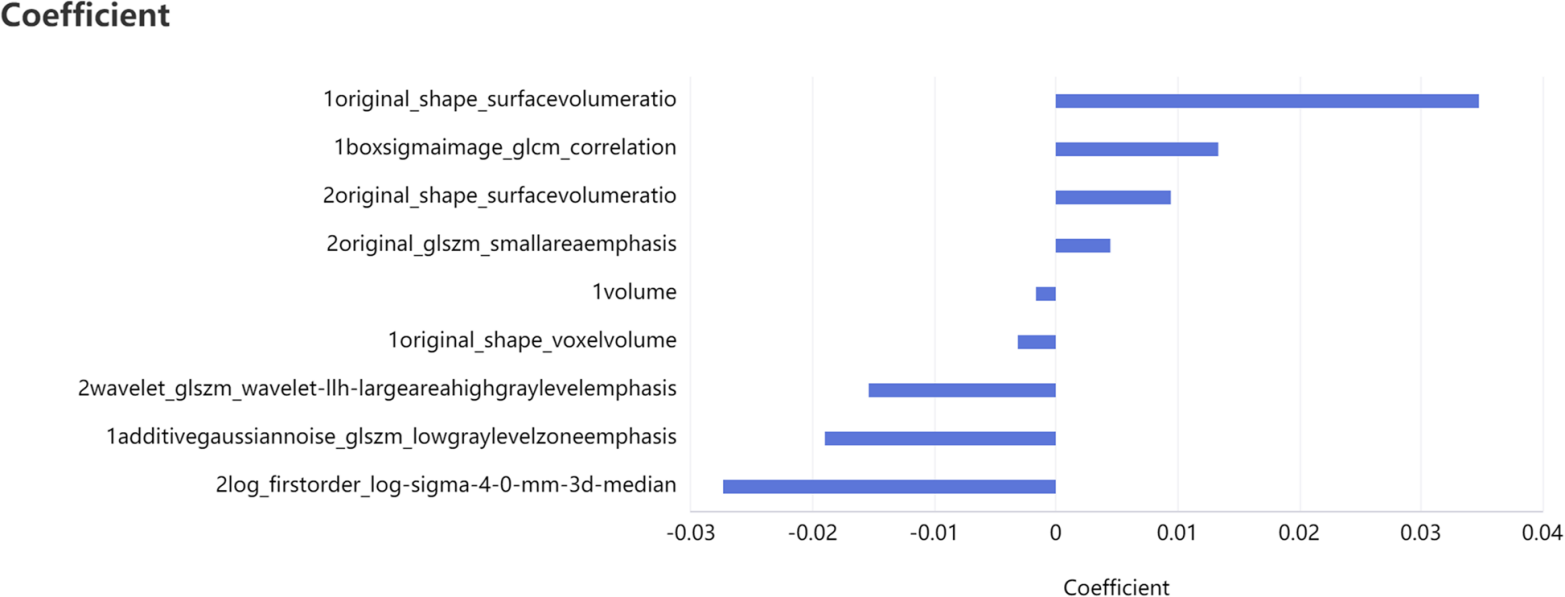
Optimal radiomic features and their coefficients

**Fig. 3:**
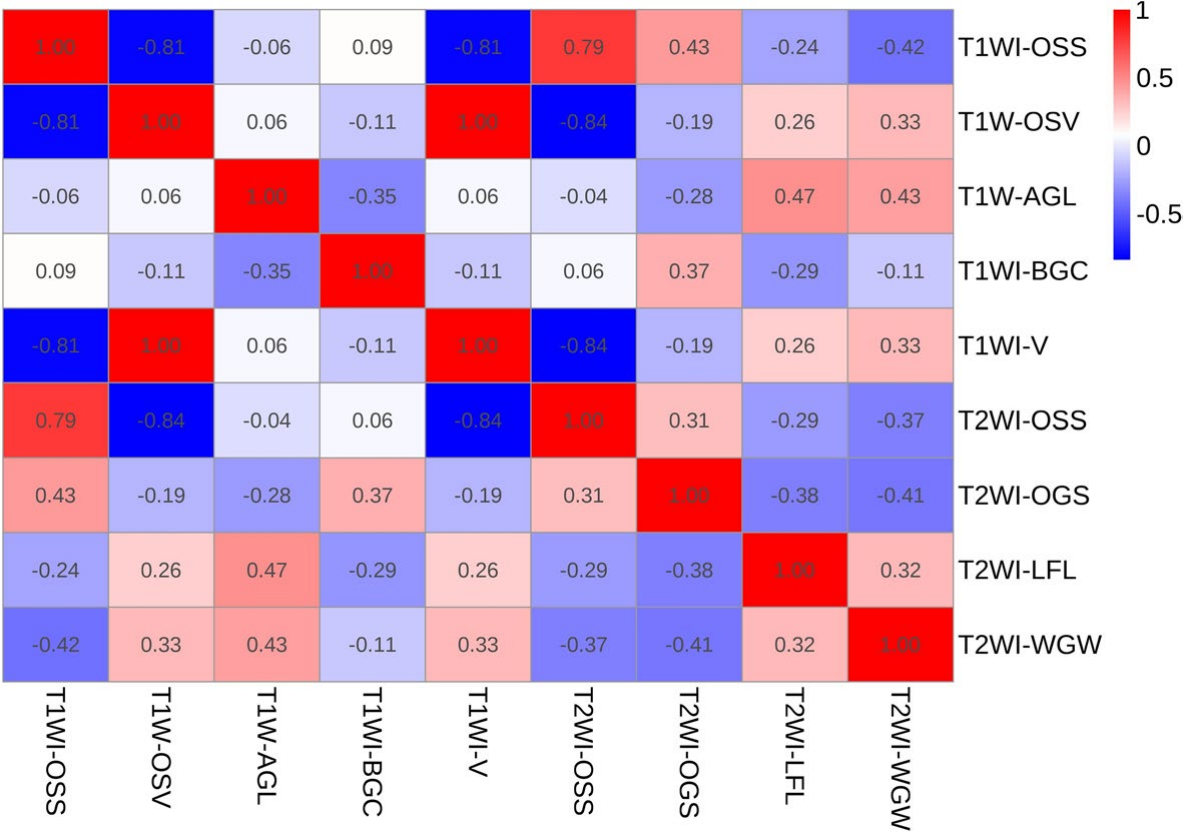
Heatmap of the correlation coefficients between optimal radiomic features

**Fig. 4:**
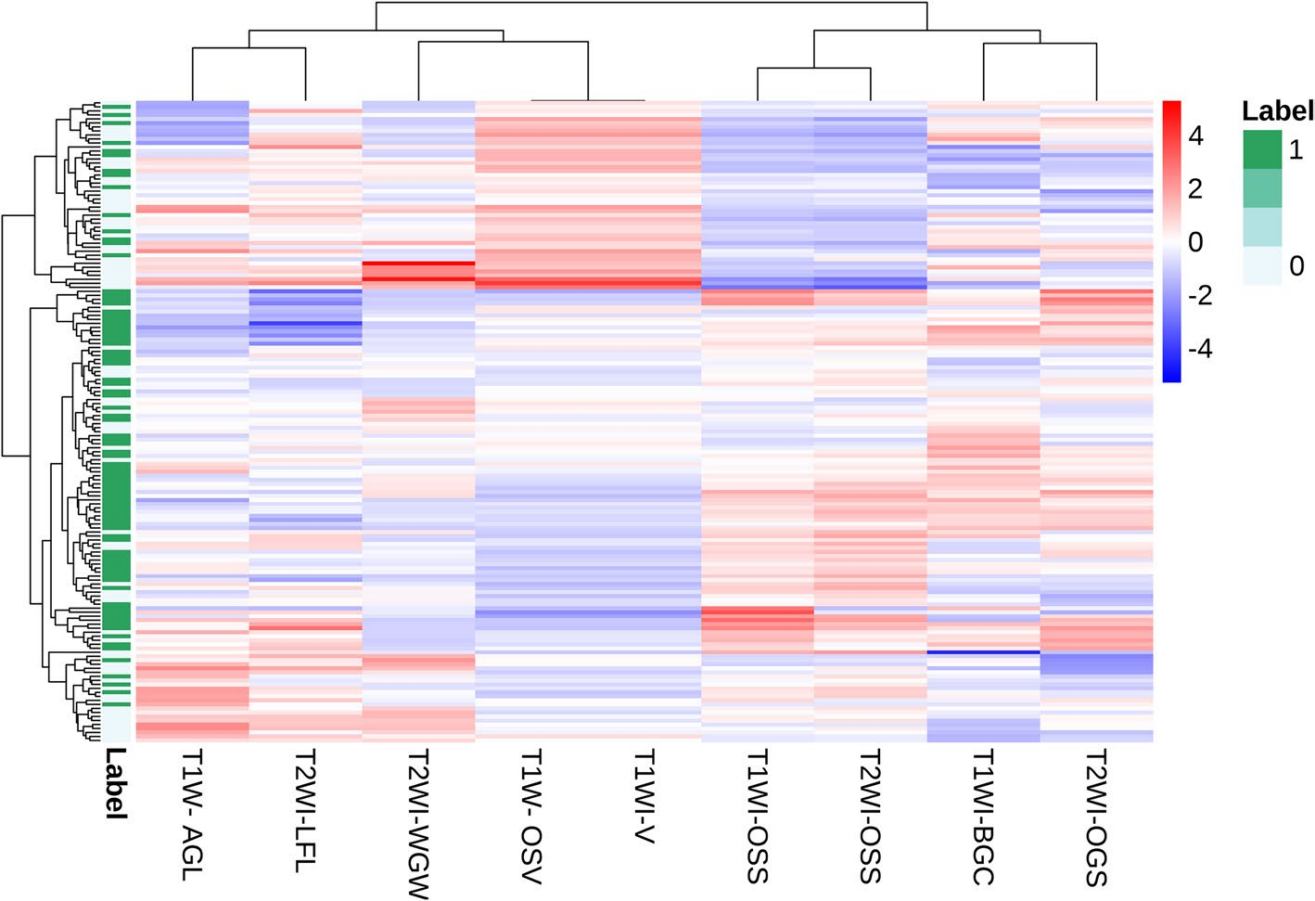
Heatmap of the optimal radiomic feature cluster analysis (label 1 indicates abnormal bone mass, label 0 indicates normal bone mass)

**Fig. 5:**
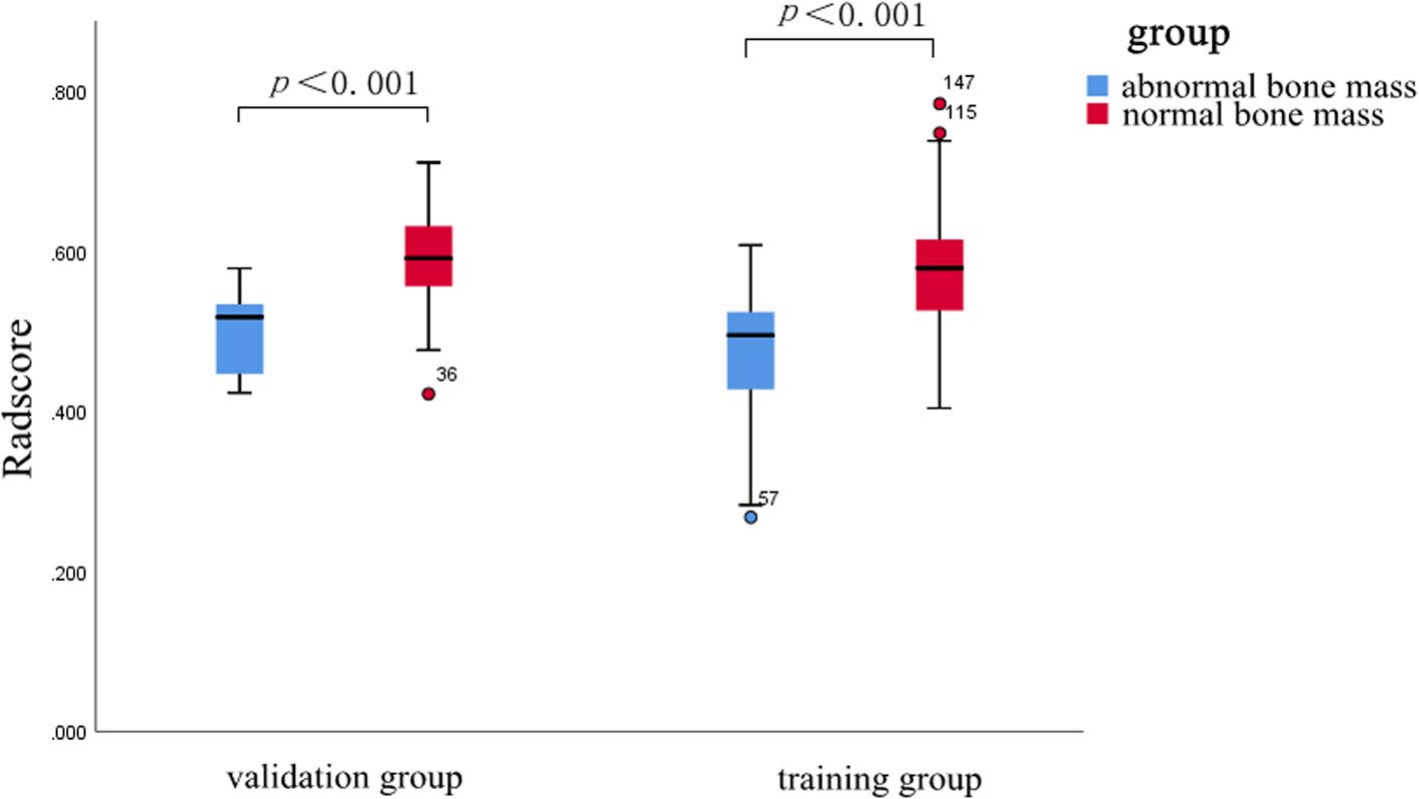
Comparison of Radscores (T1WI+T2WI) for the training group and validation group between normal and abnormal bone mass

In this study, a total of 2286 radiomic features in 8 categories were extracted, A total of five T1WI features, namely, the 1original_shape_surfacevolumeratio(T1WI-OSS), 1boxsigmaimage_glcm_correlation(T1WI-BGC),1volume(T1WI-V), 1original_shape_voxelvolume(T1WI-OSV), 1additivegaussiannoise_glszm_lowgraylevelzoneemphasis(T1WI-AGL), and four T2WI features, namely, the 2original_shape_surfacevolumeratio(T2WI-OSS), 2original_glszm_smallareaemphasis(T2WI-OGS), 2wavelet_glszm_wavelet-llh-largeareahighgraylevelemphasis(T2WI-WGW), and 2log_firstorder_log-sigma-4-0-mm-3d-medianfeatures(T2WI-LFL) were obtained by dimensionality reduction of all the features via LASSO regression(λ=0.13) (Fig. 2). The correlation coefficients between the features are shown in Fig. 3, and the heatmap of the optimal radiomic feature cluster analysis is shown in Fig. 4.Weighted summation was performed according to the coefficients of the radiomic features, including T1WI, T2WI and T1WI+T2WI to calculate the radscores. A comparison of the radscores (T1WI+T2WI) between the training group and the validation group indicated that the radscores of the patients with abnormal bone mass were significantly greater than those of the patients with normal bone mass (*P* < 0.001) (Fig. 5). The formula used was as follows:

$$\mathrm{Radscore}=0.035\ast(\mathrm T1\mathrm{WI}-\mathrm{OSS})+0.013\ast(\mathrm T1\mathrm{WI}-\mathrm{BGC})+0.009\ast(\mathrm T2\mathrm{WI}-\mathrm{OSS})+0.004\ast(\mathrm T2\mathrm{WI}-\mathrm{OGS})-0.002\ast(\mathrm T1\mathrm{WI}-\mathrm V)-0.003\ast(\mathrm T1\mathrm{WI}-\mathrm{OSV})-0.015\ast(\mathrm T2\mathrm{WI}-\mathrm{WGW})\;-0.019\ast(\mathrm T1\mathrm{WI}-\mathrm{AGL})-0.027\ast(\mathrm T2\mathrm{WI}-\mathrm{LFL})+0.538$$

**Fig. 6:**
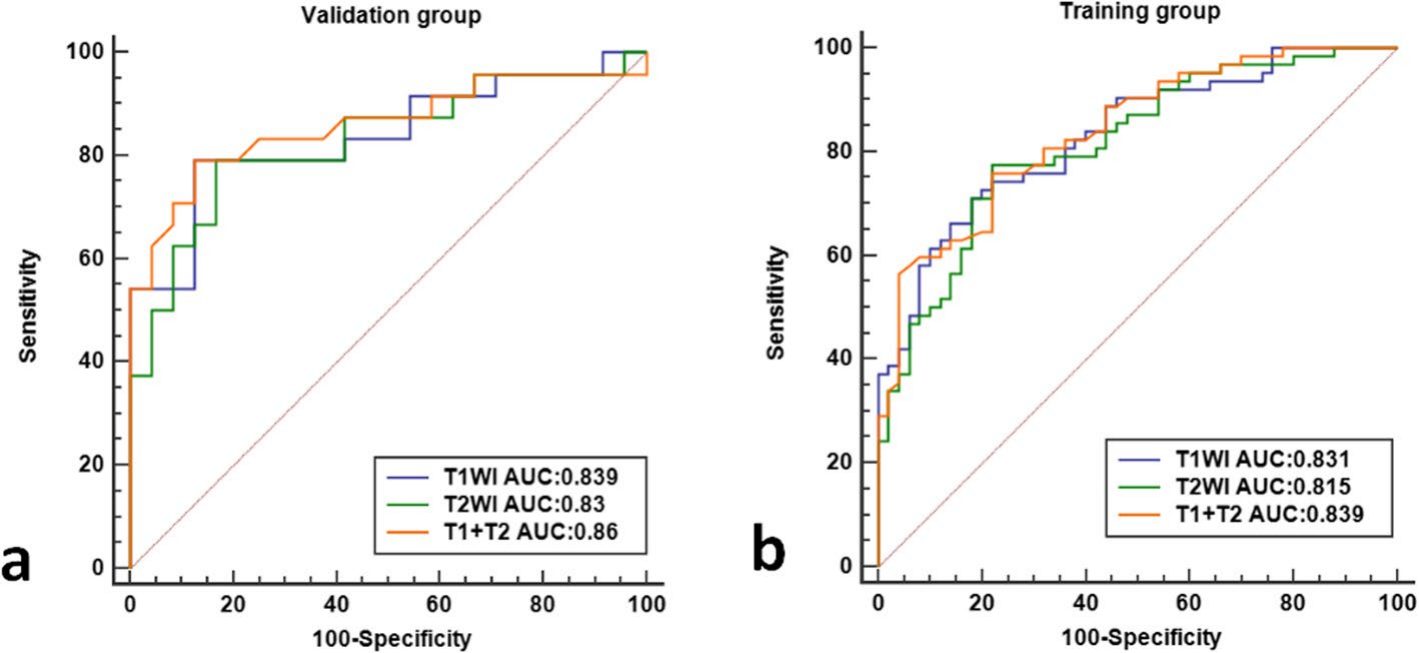
Comparison of the ROC curves of each radiomic model in the training group and the validation group.

**Fig. 7:**
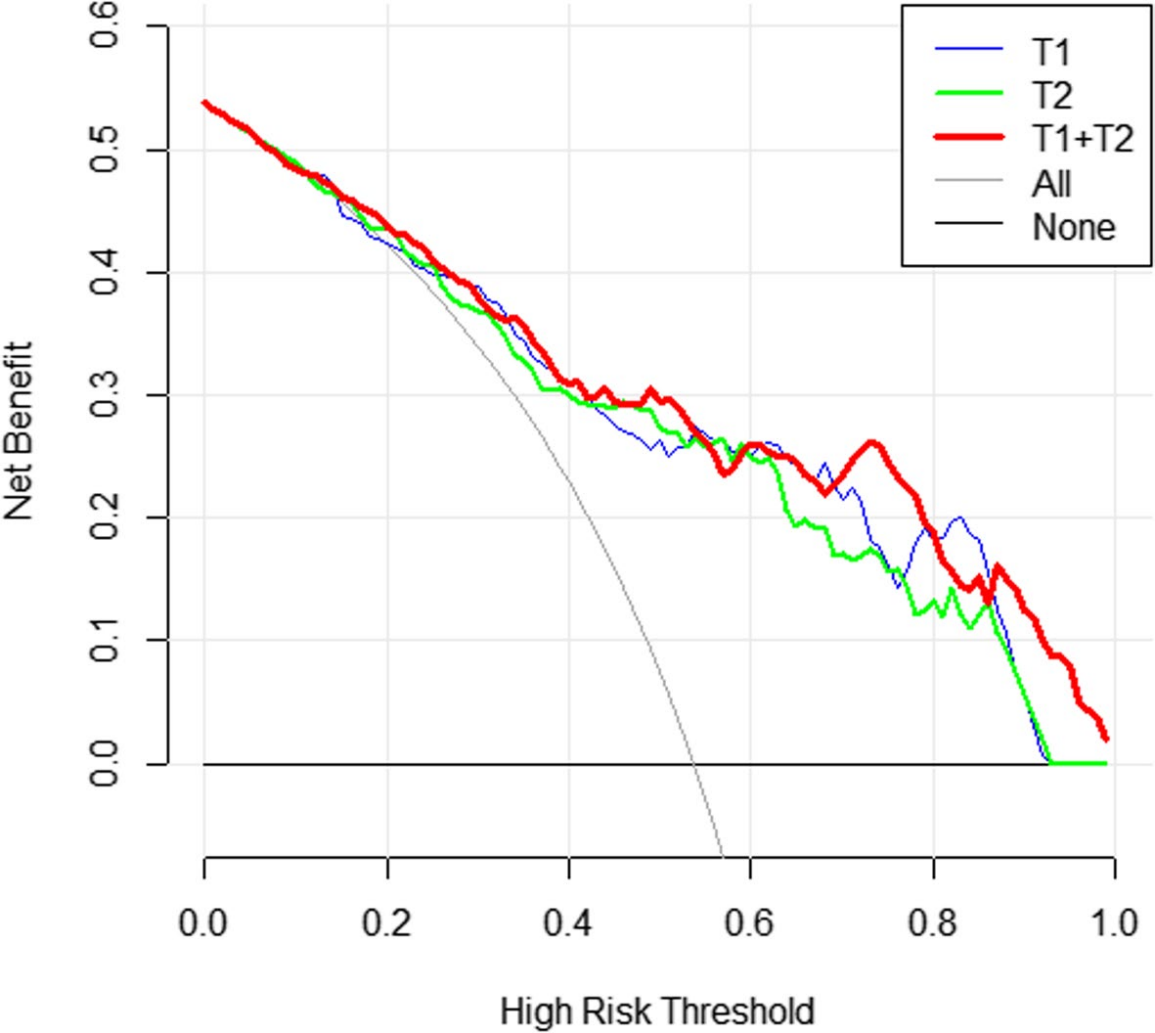
DCA of each model in the training group (the gray line representsthe assumption that all patients developed a high risk of abnormal bone mass, and the black line represents that no patient had a high risk of abnormal bone mass.) The combined model (T1+T2), which had the highest area under the curve, was the optimal decision-making model for determining the maximal net benefit in the stratification of abnormal bone mass

**Fig. 8:**
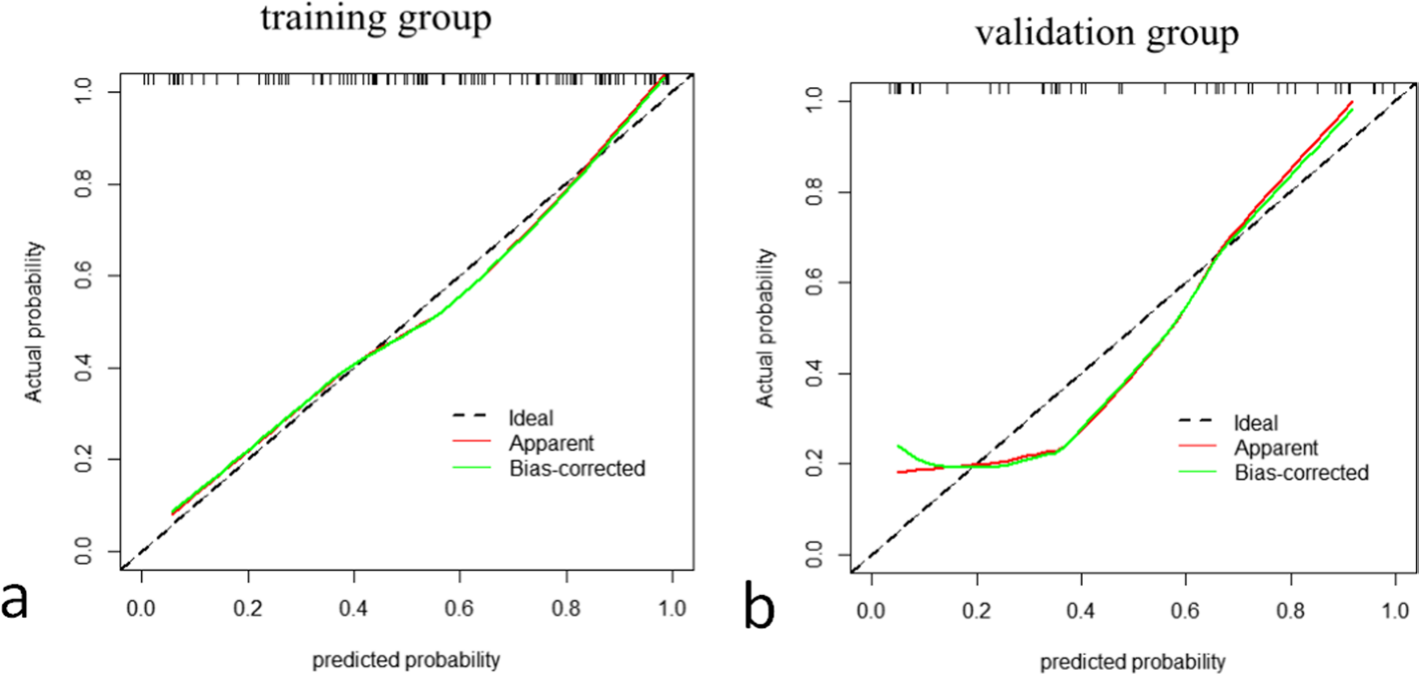
The calibration curves of the combined model

**Fig. 9:**
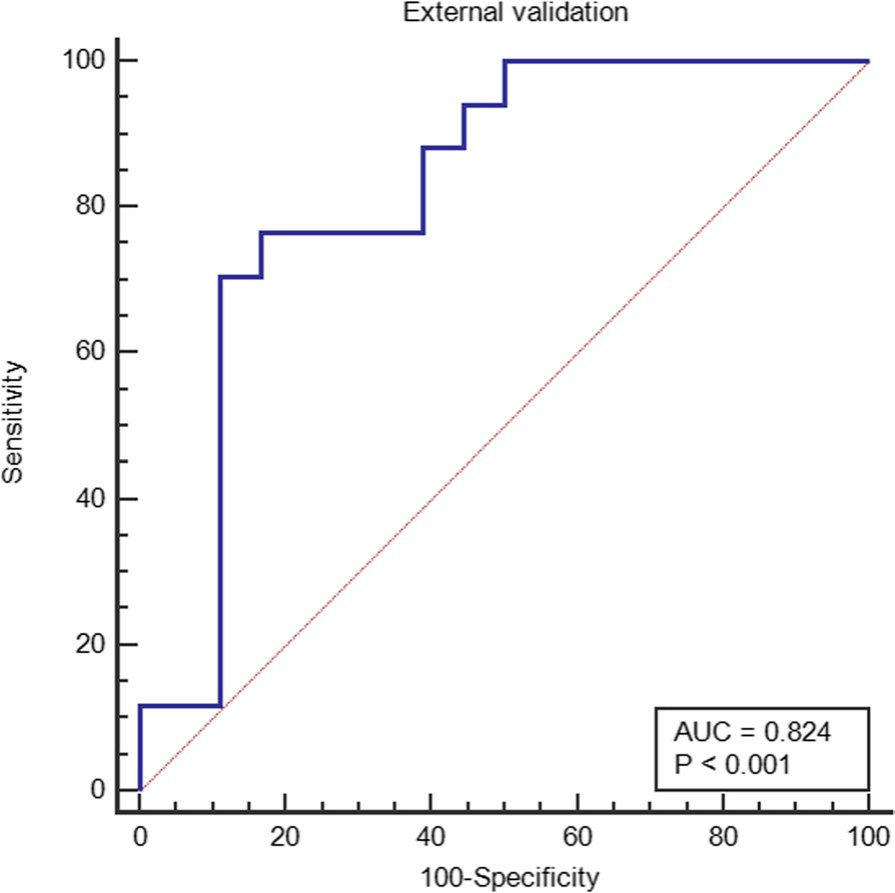
ROC curves of the radiomic model in the external validation group

A radiomic feature model for predicting lumbar bone abnormalities in patients with low back pain was constructed based on radscores. The model fit well for both the training group and the validation group(*P* > 0.05). The performance of the models was compared using ROC curves (Fig. 6) and tables (Tables 2 and 3).The AUC of the three models in the training group and validation group were compared, and the *p* values were all greater than 0.05. DCA also revealed that the combined model had better predictive performance than the single-sequence radiomic model (Fig. 7). The calibration curves of the combined model are shown in Fig. 8. The Hosmer–Lemeshow test showed that the model fit was good for both the training group (*P*= 0.93) and validation group ( *P*= 0.57). A separate external validation group was also used, and the clinical characteristics of the patients are shown in Table 4. The ROC curve of the external validation group is shown in Fig. 9, and the model performance is shown in Table 5. The results of the external validation demonstrated the good performance of the radiomic model.

**Table 2:** Performance of the radiomic model in the training group

Model	AUC(95%CI)	Cutoff	Sensitivity	Specificity	Accuracy
T1WI	0.831(0.748-0.895)	>0.598	0.71	0.82	0.760
T2WI	0.815(0.731-0.882)	>0.546	0.774	0.78	0.777
T1+T2	0.839(0.757-0.901)	>0.525	0.758	0.78	0.768

**Table 3:** Performance of radiomic model in the validation group

Model	AUC(95%CI)	Cutoff	Sensitivity	Specificity	Accuracy
T1WI	0.839(0.704-0.929)	>0.606	0.792	0.875	0.833
T2WI	0.830(0.694-0.923)	>0.578	0.792	0.833	0.813
T1+T2	0.860(0.730-0.943)	>0.542	0.792	0.875	0.833

**Table 4:** Clinical characteristics of the individuals in the external validation group

Variable	Normal(*n*=18)	Abnormal(*n*=17)	P
Age	55.3±13.9	67.1±9.3	0.006*
Gender (Male/Female)	3/15	3/14	1
Height (cm)	161±5.6	159.2±6.7	0.373
Weight (kg)	62.3±8.4	59.6±6.6	0.306
BMI	24±3.2	23.6±2.4	0.642

*Indicates that the difference was statistically significant

**Table 5:** Performance of the radiomic model in the external validation group

Model	AUC(95%CI)	Cutoff	Sensitivity	Specificity	Accuracy
T1+T2	0.824(0.678-0.969)	>0.529	0.765	0.833	0.8

## Discussion

In this study, a radiomic model based on T1 and T2-weighted images of conventional lumbar MR images was established to predict lumbar bone mass abnormalities in patients with low back pain. The results demonstrated that the combined model of T1- and T2-weighted images could accurately quantify lumbar bone mass and identify patients with abmormal bone mass. The diagnostic efficiency of the combined model was greater than that of a single-sequence model. Furthermore, the DCA also indicated that the combined model provided better net benefits to the single-sequence model. Additionally, the external validation results corroborated the high diagnostic efficiency of the model. Therefore, this method may help reduce or prevent unnecessary repeated examinations and costs associated with dual-energy radiography in the screening for osteoporosis.

Globally, various measures, such as questionnaires, primary doctor education, and medical insurance coverage, have been implemented to improve the osteoporosis screening rate [1]. However, a significant number of patients in China undergo lumbar MRI or computed tomography (CT) each year due to low back pain. Utilizing these data for osteoporosis screening could yield significant benefits. Several studies have reported optimistic findings based on this idea. The mean CT value has been found to be useful in diagnosing osteoporosis in most studies [15, 16]. There are also a few studies utilizing artificial intelligence for osteoporosis screening using X-ray and CT images. Hong, N et al. [17] demonstrated the potential of the bone radiomic score for improving hip fracture prediction by studying the texture features of DXA hip images from women with and without fractures. Lim, H K et al [1] demonstrated the high effectiveness of an abdominal CT-based radiomic model in predicting osteoporosis, with an accuracy, specificity and negative predictive value exceeding 93%. However, studies on osteoporosis screening based on MRI are rare, possibly due to the complexity of imaging and the low efficiency of manual delineation. In this study, we attempted to construct an automatic vertebral body segmentation model using deep learning methods, although the sample size was relatively small. While not all vertebral bodies were accurately segmented due to the sample size, the model consistently delineated 1-2 vertebral bodies. Suri, A et al. developed an accurate automatic segmentation model of lumbar magnetic resonance images using a deep learning algorithm based on 1123 lumbar magnetic resonance images, proving the future feasibility of this research direction [18]. In this study, the AUC for the training group and the validation group were 0.839 (0.757-0.901) and 0.860 (0.73-0.943), respectively, similar to the findings of Li He et al [19], However, their study focused only on delineating three vertebrae from the L2-L3 vertebrae as the region of interest, whereas DXA typically includes the L1-L4 vertebrae, making our results potentially more reliable. Additionally, while their study compared normal bone mass, osteopenia and osteoporosis in pairs, the selected radiomic features for each comparison were not consistent, leading to the construction of a nonuniform model that may affect the stability of the model.

A total of five radiomic features from T1WI and four radiomics features from T2WI were selected for this study. The combined model using both features proved to be more efficient than the single-sequence model. This finding suggested that both T1WI and T2WI contribute to the identifying bone abnormalities.The Shape Features (3D), including T1WI-OSS, T1WI-V, T1WI-OSV, and T2WI-OSS, indicated that changes in the shape of the vertebral body greatly influence bone mass. The gray-level size zone matrix (GLSZM) features including T1WI-AGL, T2WI-OGS, and T2WI-WGW, suggested that a change in signal strength reflect a change in bone mass. T1WI-BGC is a gray level co-occurrence matrix features that is a measure of texture fineness and roughness, and it also affects bone mass. In patients with low bone mass or osteoporosis, the trabecular bone of the vertebral body becomes thinner, the number of trabeculae decreases, the gap between trabeculae increases, the bone mass decreases, and the amount of yellow bone marrow increases [7–20]. Schwartz et al.conducted a study that showed higher levels of bone marrow fat in patients with osteoporosis, which was negatively correlated with bone formation. The yellow bone marrow is rich in hydrogen protons, and its enhancement can significantly reduce T1 relaxation times, resulting in an increase in tissue signal intensity. Therefore, the T1WI signal intensity of the vertebral body is negatively correlated with the BMD. Like these findings, T2WI can also serve as a diagnostic tool for osteoporosis in this study. Changes in the T2WI signal may be more sensitive to the changes in blood and water components [21]. Qiu, X et al. demonstrated that reduced blood microcirculation, blood flow, and inorganic components are among the causes of osteoporosis. Yellow bone marrow exhibited a slightly high signal on T2WI, and the T2WI signal increased with the increasing yellow bone content. A reduction in blood microcirculation, blood flow or combined water content can lead to a decrease in the T2WI signal [6].Thus, although changes in the T2WI signal may not be visually observed , radiomic parameters can reflect these changes. Therefore, the application of multiparametric MRI is expected to improve diagnostic efficiency. The AUC of the combined model in this study was greater than that of the single-sequence model, which is consistent with the findings of He and Li et al. These authors suggested that different sequences provide different features of information and that the information from different sequences can complement each other [19].

Research limitations and prospects: The imaging data extracted in this study were obtained through manual and semiautomatic segmentation, which is a labor-intensive task. However, an automatic segmentation model has been developed, indicating that this problem can be effectively addressed using a transfer model in the future. The sample size of this study is limited, particularly in terms of external validation data, and expanding the sample size will undoubtedly yield more valuable insights.

## Conclusions

A multiparametric MR radiomic model based on conventional T1WI and T2WI sequences was constructed in this study, this model can serve as a supplementary screening and prediction tool for osteoporosis patients. This approach provides an opportunity for early intervention and helps reduce the risk of fracture in patients.

## Data Availability

No datasets were generated or analysed during the current study.

## Abbreviations

AUC: Area under the curve
BMD: Bone mineral density
BMI: Body Mass Index
CI: Confidence intervals
CT: Computed tomography
DCA: Decision curve analysis
DICOM: Digital Imaging and Communications in Medicine
DXA: Dual-energy X-ray absorptiometry
ICC: Intraclass correlation coefficients
LASSO: Least Absolute Shrinkage and Selection Operator
MSK: Musculoskeletal
ROC: Receiver operating characteristic
ROI: Regions of interest
T1WI: T1-weighted images
T2WI: T2-weighted images
MRI: Magnetic resonance imagine
